# Correction: Microglia Display Heterogeneous Initial Responses to Disseminated Tumor Cells

**DOI:** 10.1158/0008-5472.CAN-26-2783

**Published:** 2026-08-01

**Authors:** Takahiro Tsuji, Haruka Hirose, Daisuke Sugiyama, Mariko Shindo, Rahadian Yudo Hartantyo, Yutaro Saito, Tsuyako Tatematsu, Shouta Sugio, Makoto Sanbo, Masumi Hirabayashi, Yasuhiro Kojima, Jun Koseki, Kazutaka Hosoya, Hiroshi Yoshida, Tatsuya Ogimoto, Yuto Yasuda, Kentaro Hashimoto, Hitomi Ajimizu, Yuichi Sakamori, Hironori Yoshida, Noritaka Sano, Masahiro Tanji, Hiroaki Ito, Kazuhiro Terada, Masatsugu Hamaji, Toshi Menju, Hiroyuki Konishi, Shogo Kumagai, Cyrus M. Ghajar, Daisuke Kato, Hiroshi Date, Akihiko Yoshizawa, Yoshiki Arakawa, Hiroaki Ozasa, Andrew J. Moorhouse, Teppei Shimamura, Hiroyoshi Nishikawa, Toyohiro Hirai, Hiroaki Wake

In the original version of this article (1), author Katsuaki Sato was mistakenly omitted from the author list. This error has been corrected in the latest online HTML and PDF versions of the article and the author contributions and disclosures have been updated as necessary. The authors regret this error.
